# Complete Genome Sequence of Methanofollis formosanus DSM 15483^T^, Isolated from an Aquaculture Fish Pond

**DOI:** 10.1128/mra.00068-22

**Published:** 2022-04-28

**Authors:** Sheng-Chung Chen, Chih-Hung Wu, Yi-Ting You, Sue-Yao Wu, Ching-Hua Liao, Xiang Wang, Hongduo Li, Yun Guo, Jinhui You, Wanling Qiu

**Affiliations:** a School of Resources and Chemical Engineering, Sanming University, Sanming, Fujian, People’s Republic of China; b Fujian Provincial Key Laboratory of Resources and Environmental Monitoring and Sustainable Management and Utilization, Sanming University, Sanming, Fujian, People’s Republic of China; c Department of Life Sciences, National Chung Hsing University, Taichung, Taiwan, Republic of China; d College of Environment & Safety Engineering, Fuzhou University, Fuzhou, Fujian, People’s Republic of China; Montana State University

## Abstract

The hydrogenotrophic strain Methanofollis formosanus DSM 15483^T^ (= ML15^T^ = OCM 798^T^) was isolated from an aquaculture fish pond near Wang-gong, Taiwan. The genome of strain DSM 15483^T^ was selected for sequencing in order to provide further information about the species delineation and its unique habitat.

## ANNOUNCEMENT

At present, a total of 6 *Methanofollis* species have been characterized and validly described: Methanofollis tationis (1, 2), M. liminatans (2, 3), M. aquaemaris (4), M. formosanus (5), M. ethanolicus (6), and M. fontis (7). Isolates of the genus *Methanofollis* are widespread in various anaerobic environments, such as solfataric fields, wastewater reactors, aquaculture fish ponds, lotus fields, and cold seep sediments (1, 3–7). Moreover, *Methanofollis*-related sequences have been found in acetate-rich gas-petroleum reservoir surface facilities (8), municipal solid waste landfill leachates (9), a long-duration gas injection oil reservoir in the south of Iran (10), a crust formed on SS400 carbon steel during corrosion (11), and biogas reactors (12, 13). Here, we report the complete genome sequence of *M. formosanus* DSM 15483^T^ to further understand the microbial adaptation to various environments.

*M. formosanus* DSM 15483^T^ (= ML15^T^ = OCM 789^T^) was obtained from the Leibniz Institute DSMZ, grown in anaerobic MB/W medium with 100 mM sodium formate and 5 mM sodium acetate, and incubated at 37°C, according to the method used in our previous studies (5, 14, 15). Genomic DNA from strain DSM 15483^T^ was isolated using a modification of the methods of Jarrell et al. (16) and Johnson (17). Briefly, cells from 500 mL of culture were lysed with sodium dodecyl sulfate (SDS) (1%, wt/vol). After phenol-chloroform extraction and ethanol precipitation, the quantity and quality of the dissolved DNA samples were examined using a UV-visible (UV-Vis) spectrophotometer.

The genome was sequenced at the Genomics BioSci and Tech Co., Ltd. (Taiwan), using the Illumina MiSeq platform. The genomic DNA was sheared randomly, and a paired-end DNA library of 300-bp reads was constructed using the TruSeq Nano DNA high-throughput (HT) library prep kit and the TruSeq DNA kit with 96 CD indexes (Illumina). The constructed DNA library was sequenced using the MiSeq reagent kit v3 (600 cycle) on the MiSeq platform (Illumina), and 300-bp paired-end reads (~1.79 Gb) were generated by the Genomics BioSci and Tech Co. All generated reads were quality trimmed to obtain high-quality reads using Trimmomatic (18). These reads were *de novo* assembled using SPAdes v3.10.1 (19), and the quality of the assembled genome was evaluated using QUAST v4.5 (20). The sequencing protocol generated 337× mean coverage of the genome. The longest contig obtained comprised an *N*_50_ value of 2,966,023 bp and was circularized by aligning both ends of the contig sequences (~300 bp) and deleting the overlapping sequences from one end. The genes of the genome were identified using the Prokaryotic Genome Annotation Pipeline (PGAP) at the website of the National Center for Biotechnology Information (http://www.ncbi.nlm.nih.gov) (21).

The complete genome of strain DSM 15483^T^ comprised a total of 2,965,921 bp and an average G+C content of 60.32%. No plasmids were identified. The genome was predicted to harbor 2,676 genes, of which 2,568 were protein coding. The genome contains 4 rRNA genes and 51 tRNA genes. Two clustered regularly interspaced short palindromic repeats (CRISPRs) with a high evidence level were found in the genome using CRISPRCasFinder (22). Default parameters were used for all bioinformatics analyses.

### Data availability.

This whole-genome shotgun project has been deposited at DDBJ/ENA/GenBank under accession number CP037968.1. The version described in this paper is the first version. The BioProject accession number is PRJNA527005. The raw sequence reads have been deposited in the Sequence Read Archive (SRA) under accession number SRR17713754.

## References

[1] Zabel HP, König H, Winter J. 1984. Isolation and characterization of a new coccoid methanogen, *Methanogenium tatii* spec. nov. from a solfataric field on Mount Tatio. Arch Microbiol 137:308–315. doi:10.1007/BF00410727.

[2] Zellner G, Boone DR, Keswani J, Whitman WB, Woese CR, Hagelstein A, Tindall B, Stackebrandt E. 1999. Reclassification of *Methanogenium tationis* and *Methanogenium liminatans* as *Methanofollis tationis* gen. nov., comb. nov. and *Methanofollis liminatans* comb. nov. and description of a new strain of *Methanofollis liminatans*. Int J Syst Evol Microbiol 49:247–255. doi:10.1099/00207713-49-1-247. 10028269

[3] Zellner G, Sleytr UB, Messner P, Kneifel H, Winter J. 1990. *Methanogenium liminatans* spec. nov., a new coccoid, mesophilic methanogen able to oxidize secondary alcohols. Arch Microbiol 153:287–293. doi:10.1007/BF00249084.

[4] Lai MC, Chen SC. 2001. *Methanofollis aquaemaris* sp. nov., a methanogen isolated from an aquaculture fish pond. Int J Syst Evol Microbiol 51:1873–1880. doi:10.1099/00207713-51-5-1873. 11594621

[5] Wu SY, Chen SC, Lai MC. 2005. *Methanofollis formosanus* sp. nov., isolated from a fish pond. Int J Syst Evol Microbiol 55:837–842. doi:10.1099/ijs.0.63475-0. 15774671

[6] Imachi H, Sakai S, Nagai H, Yamaguchi T, Takai K. 2009. *Methanofollis ethanolicus* sp. nov., an ethanol-utilizing methanogen isolated from a lotus field. Int J Syst Evol Microbiol 59:800–805. doi:10.1099/ijs.0.003731-0. 19329610

[7] Chen SC, Teng NH, Lin YS, Lai MC, Chen HH, Wang CC. 2020. *Methanofollis fontis* sp. nov., a methanogen isolated from marine sediment near a cold seep at Four-Way Closure Ridge offshore southwestern Taiwan. Int J Syst Evol Microbiol 70:5497–5502. doi:10.1099/ijsem.0.004440. 32897849

[8] Shimizu S, Ueno A, Ishijima Y. 2011. Microbial communities associated with acetate-rich gas-petroleum reservoir surface facilities. Biosci Biotechnol Biochem 75:1835–1837. doi:10.1271/bbb.110243. 21897018

[9] Laloui-Carpentier W, Li T, Vigneron V, Mazéas L, Bouchez T. 2006. Methanogenic diversity and activity in municipal solid waste landfill leachates. Antonie Van Leeuwenhoek 89:423–434. doi:10.1007/s10482-005-9051-9. 16779637

[10] Pournia M, Bahador N, Salekdeh GH. 2018. Microbial diversity of long-duration gas injection oil reservoir based on next generation sequencing in south of Iran. Nat Environ Pollut Technol 17:413–420.

[11] Hirano S-I, Nagaoka T, Matsumoto N. 2020. Microbial community dynamics in a crust formed on carbon steel SS400 during corrosion. Corros Eng Sci Technol 55:685–692. doi:10.1080/1478422X.2020.1774961.

[12] Karabey B, Daglioglu ST, Azbar N, Ozdemir G. 2019. Bacterial and archaeal dynamics of a labscale HYBRID gas fermentation bioreactor fed with CO_2_ and H_2_. J Environ Sci Health A Tox Hazard Subst Environ Eng 54:1348–1355. doi:10.1080/10934529.2019.1649589. 31446840

[13] Zhao C, Ai C, Li Q, Yang C, Zhou G, Liu B. 2017. Diversity of archaea and bacteria in a biogas reactor fed with *Pennisetum sinese* Roxb by 16S rRNA sequence analysis. Trop J Pharm Res 15:2659–2667. doi:10.4314/tjpr.v15i12.18.

[14] Weng C-Y, Chen S-C, Lai M-C, Wu S-Y, Lin S, Yang TF, Chen P-C. 2015. *Methanoculleus taiwanensis* sp. nov., a methanogen isolated from deep marine sediment at the deformation front area near Taiwan. Int J Syst Evol Microbiol 65:1044–1049. doi:10.1099/ijs.0.000062. 25575827

[15] Chen SC, Chen MF, Lai MC, Weng CY, Wu SY, Lin S, Yang TF, Chen PC. 2015. *Methanoculleus sediminis* sp. nov., a methanogen from sediments near a submarine mud volcano. Int J Syst Evol Microbiol 65:2141–2147. doi:10.1099/ijs.0.000233. 25855623

[16] Jarrell KF, Faguy D, Hebert AM, Kalmokoff ML. 1992. A general method of isolating high molecular weight DNA from methanogenic archaea (archaebacteria). Can J Microbiol 38:65–68. doi:10.1139/m92-010. 1316221

[17] Johnson JL. 1985. DNA reassociation and DNA hybridization of bacterial nucleic acids. Methods Microbiol 18:33–74.

[18] Bolger AM, Lohse M, Usadel B. 2014. Trimmomatic: a flexible trimmer for Illumina sequence data. Bioinformatics 30:2114–2120. doi:10.1093/bioinformatics/btu170. 24695404 PMC4103590

[19] Bankevich A, Nurk S, Antipov D, Gurevich AA, Dvorkin M, Kulikov AS, Lesin VM, Nikolenko SI, Pham S, Prjibelski AD, Pyshkin AV, Sirotkin AV, Vyahhi N, Tesler G, Alekseyev MA, Pevzner PA. 2012. SPAdes: a new genome assembly algorithm and its applications to single-cell sequencing. J Comput Biol 19:455–477. doi:10.1089/cmb.2012.0021. 22506599 PMC3342519

[20] Gurevich A, Saveliev V, Vyahhi N, Tesler G. 2013. QUAST: quality assessment tool for genome assemblies. Bioinformatics 29:1072–1075. doi:10.1093/bioinformatics/btt086. 23422339 PMC3624806

[21] Tatusova T, DiCuccio M, Badretdin A, Chetvernin V, Nawrocki EP, Zaslavsky L, Lomsadze A, Pruitt KD, Borodovsky M, Ostell J. 2016. NCBI Prokaryotic Genome Annotation Pipeline. Nucleic Acids Res 44:6614–6624. doi:10.1093/nar/gkw569. 27342282 PMC5001611

[22] Couvin D, Bernheim A, Toffano-Nioche C, Touchon M, Michalik J, Néron B, Rocha EP, Vergnaud G, Gautheret D, Pourcel C. 2018. CRISPRCasFinder, an update of CRISRFinder, includes a portable version, enhanced performance and integrates search for Cas protens. Nucleic Acids Res 46:W246–W251. doi:10.1093/nar/gky425. 29790974 PMC6030898

